# Caveolin-1-Knockout Mouse as a Model of Inflammatory Diseases

**DOI:** 10.1155/2018/2498576

**Published:** 2018-08-29

**Authors:** Elena Codrici, Lucian Albulescu, Ionela Daniela Popescu, Simona Mihai, Ana-Maria Enciu, Radu Albulescu, Cristiana Tanase, Mihail E. Hinescu

**Affiliations:** ^1^Victor Babes National Institute of Pathology, 050096 Bucharest, Romania; ^2^Carol Davila University of Medicine and Pharmacy, 050047 Bucharest, Romania; ^3^National Institute for Chemical Pharmaceutical R&D, Bucharest, Romania; ^4^Faculty of Medicine, Titu Maiorescu University, Bucharest, Romania

## Abstract

Caveolin-1 (CAV1) is the scaffold protein of caveolae, which are minute invaginations of the cell membrane that are involved in endocytosis, cell signaling, and endothelial-mediated inflammation. CAV1 has also been reported to have a dual role as either a tumor suppressor or tumor promoter, depending on the type of cancer. Inflammation is an important player in tumor progression, but the role of caveolin-1 in generating an inflammatory milieu remains poorly characterized. We used a caveolin-1-knockout (CAV1^−/−^) mouse model to assess the inflammatory status via the quantification of the pro- and anti-inflammatory cytokine levels, as well as the ability of circulating lymphocytes to respond to nonspecific stimuli by producing cytokines. Here, we report that the CAV1^−/−^ mice were characterized by a low-grade systemic proinflammatory status, with a moderate increase in the IL-6, TNF-*α*, and IL-12p70 levels. CAV1^−/−^ circulating lymphocytes were more prone to cytokine production upon nonspecific stimulation than the wild-type lymphocytes. These results show that CAV1 involvement in cell homeostasis is more complex than previously revealed, as it plays a role in the inflammatory process. These findings indicate that the CAV1^−/−^ mouse model could prove to be a useful tool for inflammation-related studies.

## 1. Introduction

A low-grade chronic inflammatory status is defined as a persistent, mild increase (2 to 4 times greater than normal) in circulating inflammation mediators [1]. Chronic inflammation is associated with a plethora of conditions, including aging (inflammaging) [2], autoimmune diseases [3], cardiovascular pathologies [4, 5], and carcinogenesis [6], as well as the formation and maintenance of a (pre)metastatic niche [7]. Systemic contributors to chronic inflammation are endothelial cells and immune cells, which are also now recognized as important players in tumorigenesis and metastasis [8, 9]. Caveolin-1 (CAV1), the scaffold protein of caveolae, could represent a link between inflammation and tumorigenesis, as it has been associated with both processes. In addition to its scaffolding role, CAV1 acts as a “guardian” by selecting the messages that are sent into cells from the outer environment. CAV1 recruits various cytoplasmic proteins involved in cell signaling via its caveolin-scaffolding domain. Loss of CAV1 has been associated with a proinflammatory status in senescent endothelial cells [10] and with premature senescence in fibroblasts [11] and was protumorigenic for selected cancers, such as prostate [12] and gastric [13] cancer and glioblastoma [14]. Loss of CAV1 in stromal cells, most notably in the cancer-associated fibroblasts, negatively affected the relapse-free survival of prostate cancer [15], breast cancer [16], and gastric cancer [17] patients. However, whether the lack of CAV1 is directly correlated with chronic inflammation has been insufficiently explored. The involvement of CAV1 in inflammation has only been sporadically addressed, with reports mainly focused on the evaluation of endothelial cells and their role in atherosclerosis [18, 19] and the lung response to sepsis [20–22]. For immune cells involved in the production of inflammatory mediators, CAV1 has seldom been reported as related to lymphocyte migration [23, 24] and the inhibition of proinflammatory cytokine production in macrophages [18].

The aim of this study was to specifically address the hypothesis that the loss of CAV1 is involved in the pathogeny of the inflammatory response. We examined more than 30 pro- and anti-inflammatory cytokines in the plasma of CAV1^−/−^ mice to assess their inflammatory status, as well as the ability of circulating leukocytes to respond to nonspecific stimuli through the production of cytokines.

## 2. Materials and Methods

### 2.1. Mice and Sample Collection

Blood samples were obtained from CAV1^−/−^ mice (CAV1 KO: CAV1^tm1Mls^/J) and CAV1^+/+^ mice (B6129PF2/J), purchased from Jackson Laboratory (Bar Harbor, ME) (*n* = 9). For this study, we used 3-month-old male knockout mice weighing 22 ± 4 g and age-, gender-, and weight-matched control mice. All animal experiments were conducted in accordance with the respective animal welfare guidelines, the *Guide for the Care and Use of Laboratory Animals* published by the US National Institutes of Health, and the study was approved by the Institutional Ethical Committee of “Victor Babes” National Institute of Pathology in Bucharest. The adult mice were fed with standard chow and water ad libitum.

### 2.2. Plasma Preparation

Collection of whole peripheral blood from knockout mice, the STOCK CAV1^tm1Mls^/J and control B6129PF2/J mice, was achieved using vacuum blood tubes (Systems, Becton Dickinson) with heparin (for cell culture/plasma). For plasma extraction, the blood was allowed to clot for at least 30 min at room temperature before centrifugation at 2500 rpm for 10 min. Samples were then aliquoted and stored at −80°C until the multiplex analyses. The plasma samples were collected from mice in a consistent manner, at the same time of the day, between 10:00 a.m. and 11:00 a.m.

### 2.3. Cell Culture

Whole peripheral blood of both the CAV1^−/−^ and control mice was obtained through retroorbital blood collection and diluted to 5% with RPMI-1640 culture medium (supplemented with 1% antibiotic), in the absence and presence of the polyclonal lymphocyte stimulator, 5 mg/L PHA (Difco, Augsburg, Germany), or 5 mg/L ConA (Difco, Augsburg, Germany) [25]. Whole-blood cell culture was performed in 96-well round bottom plates (Corning CLS3360); after the indicated exposure time to compounds, the plates were centrifuged at 250*g* for 10 minutes and 100 *μ*L supernatants from each sample was collected and stored in 1.8 mL cryo tubes. Cultures were incubated for 24 h and 48 h at 37°C and 5% CO_2_ (Shell Lab). Samples were made in triplicate. After 24 h or 48 h of treatment, the supernatant was removed, following centrifugation for 5 min at 250*g*. Samples were stored at −80°C until further analysis.

### 2.4. Assessment of Cytokines by xMAP Analysis

The xMAP array was performed according to the manufacturer's protocols, and the plates were analyzed using a Luminex® 200™ system (Luminex, Austin, TX). Cell culture cytokine levels were determined using the Fluorokine MAP Mouse Base Kit (R&D Systems, USA), with the following analyte-specific bead sets: *GM-CSF*, *IL-1β*, *IL-2*, *IL-4*, *IL-5*, *IL-6*, *IL-10*, *IL-12p70*, *IL-13*, *IL-17*, *CCL2/JE/MCP-1*, *CXCL1/KC*, *MIP-2*, *TNF-α*, and *VEGF*. Plasma cytokine levels were determined using the MILLIPLEX MAP Cytokine Magnetic Bead Panel Kit—*GM-CSF*, *IL-1β*, *IL-2*, *IL-4*, *IL-6*, *IL-12p70*, *IL-13*, *CXCL1/KC*, *VEGF*, and *TNF-α* (Merck-Millipore, Billerica, MA, USA). Briefly, the beads were incubated with the samples, buffers, and standards in a 96-well plate at 4°C overnight. All further incubations with the detection antibodies and streptavidin phycoerythrin (SAPE) conjugate were performed at room temperature in the dark with shaking at 800 rpm. Multiplex data acquisition and analysis were performed using STarStation 2.3 (Applied Cytometry Systems, Sheffield, UK) and xPONENT 3.1 software (Millipore, Billerica, MA); the calibration curves were generated with a 5-parameter logistic fit.

### 2.5. Proteome Profiler™ Antibody Array: A Membrane-Based Assay

Array images were scanned with MicroChemi 4.2 (Berthold Technologies, Chennai, India), and the signal intensity of each spot was analyzed with ImageJ software; the average intensity was calculated by subtracting the average background signal. The cytokine profile assessment, including *CXCL13/BLC/BCA-1*, *IL-5*, *M-CSF*, *C5a*, *IL-6*, *CCL2/JE/MCP-1*, *G-CSF*, *IL-7*, *CCL12/MCP-5*, *GM-CSF*, *IL-10*, *CXCL9/MIG*, *CCL1/I-309*, *IL-12 p70*, *CCL3/MIP-1α*, *CCL11/eotaxin*, *IL-13*, *CCL4/MIP-1β*, *ICAM-1*, *IL-16*, *CXCL2/MIP-2*, *IFNγ*, *IL-17*, *CCL5/RANTES*, *IL-1α*, *IL-23*, *CXCL12/SDF-1*, *IL-1β*, *IL-27*, *CCL17/TARC*, *IL-1ra*, *CXCL10/IP-10*, *TIMP-1*, *IL-2*, *CXCL11/I-TAC*, *TNF-α*, *IL-3*, *CXCL1/KC*, *TREM-1*, and *IL-4*, was performed using Mouse Cytokine Array Panel A (R&D Systems Inc., Minneapolis, MN, USA), according to the manufacturer's instructions. Briefly, after the membrane blocking, the plasma samples and detection antibody cocktail were added and incubated overnight at 4°C on a rocking platform shaker. After the unbound proteins were removed by washing, the membranes were incubated with a streptavidin-HRP solution for 30 min at room temperature on a rocking platform and then washed again. Subsequently, protein spots were visualized using the chemiluminescence detection reagents.

### 2.6. Statistical Analysis

Data were expressed as mean ± standard error of the mean (SEM), and minimum and maximum values were provided when necessary. Duplicate/triplicate samples were used for all specimens, and the average concentrations were used for statistical analysis. Differences between groups were analyzed by two-tailed unpaired Student's *t*-test. Statistical significance has been indicated as ^∗^*p* < 0.05, ^∗∗^*p* < 0.01, or ^∗∗∗^*p* < 0.001. Statistical analysis was performed using GraphPad Software.

## 3. Results

In order to evaluate the inflammatory status of the CAV1^−/−^ mice, we assessed the levels of circulating pro- and anti-inflammatory cytokines and growth factors in plasma. The pattern of the cytokine and growth factor production in the CAV1^−/−^ mice, compared to the controls, was evaluated by two different multiplex analyses: xMAP technology and proteome profiler analysis. We examined adult animals, for which we confirmed the lack of CAV1 expression, before the onset of any macroscopic tumors (data not shown), in order to discriminate between existing pretumor inflammation and a tumor-driven inflammatory milieu, as these mice have been reported to be prone to tumorigenesis [26].

### 3.1. Increased Levels of IL-6, TNF-*α*, and IL-12p70 in the Plasma of CAV1^−/−^ Mice

**Figure 1:**
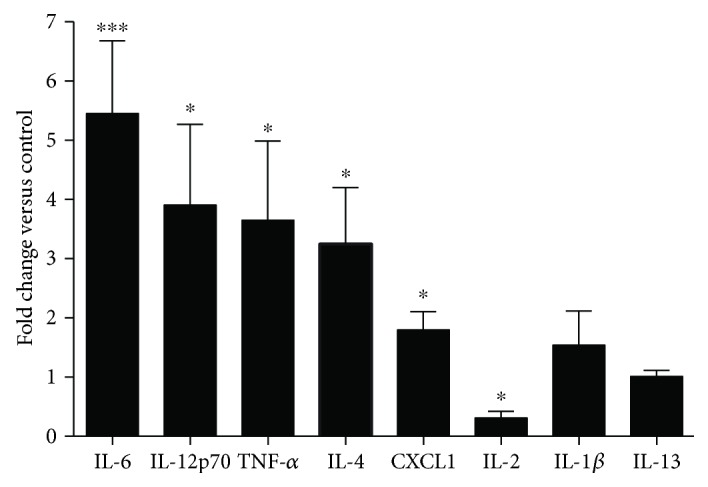
Cytokine levels in the plasma of the KO mice (*n* = 9) compared to the controls (*n* = 9). The data represent the average fold modification ± SEM versus the controls, as determined by the xMAP multiplex assay; molecules have been arranged in order of statistical significance. ^∗^*p* < 0.05 and ^∗∗∗^*p* < 0.001 indicate statistical significance compared with the controls.

Overexpression of the plasma levels of the proinflammatory cytokines IL-6 (over a 5-fold change in the KO mice versus control, *p* < 0.001) and TNF-*α* and IL-12p70 (over a 3-fold change, *p* < 0.05) was detected using the xMAP Luminex 200 platform. IL-4, as well as CXCL1/KC, was also found to be upregulated in the KO mice compared to the control mice (over a 3-fold change and up to 2-fold change, resp.; *p* < 0.05) (Figure 1).

### 3.2. Proteome Profiler Analysis of Proinflammatory Cytokines and Chemokines in the Plasma of CAV1^−/−^ Mice

**Figure 2:**
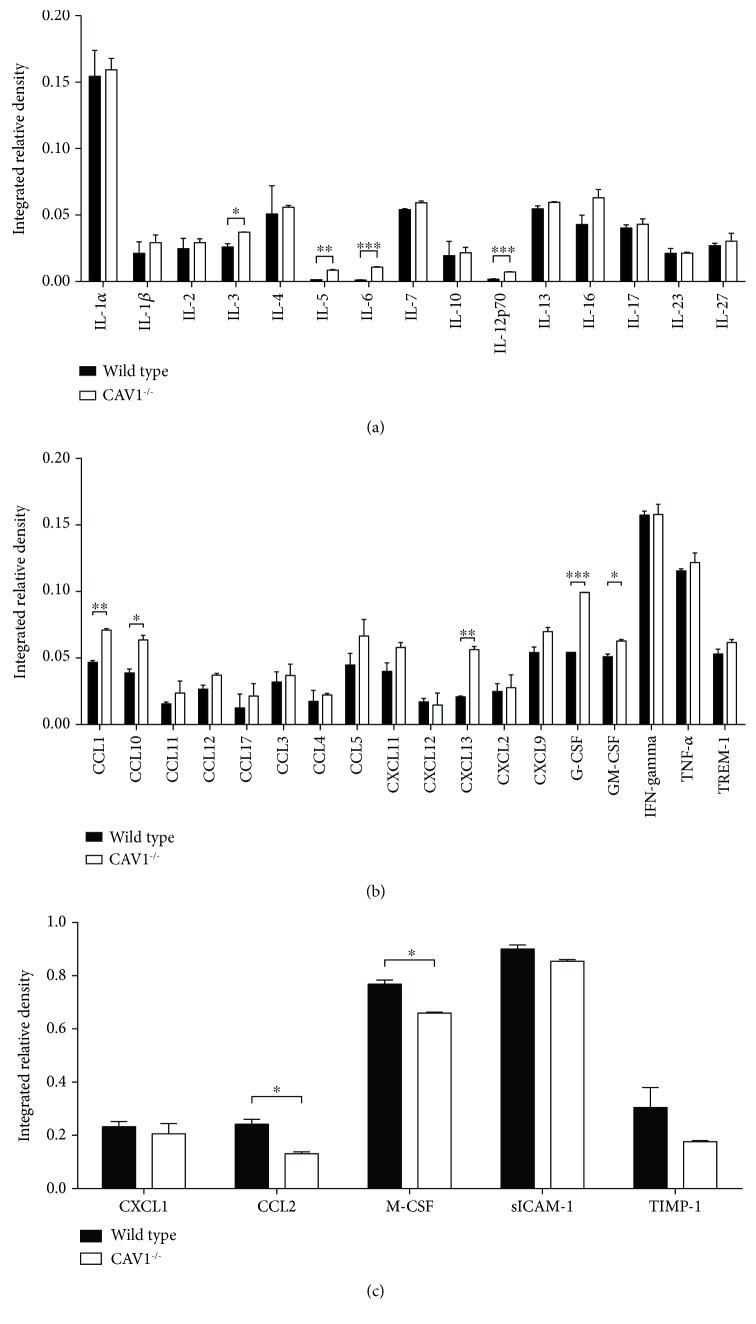
Relative expression levels of various mediators of inflammation. Pro- and anti-inflammatory cytokines (a), growth factors (b), and enzymes (c) involved in inflammatory processes were assessed in the plasma of the CAV1^−/−^ and control mice using the Proteome Profiler. The data represent the average of the experiments. ^∗^*p* < 0.05, ^∗∗^*p* < 0.01, and ^∗∗∗^*p* < 0.001.

In order to establish an overall perspective of the inflammatory status of the CAV1^−/−^ mice, we also performed an array analysis of multiple circulating pro- and anti-inflammatory cytokines and growth factors in plasma. A dot blot assay revealed a relevant pattern for the proinflammatory status (Figures 2(a) and 2(c)). The results showed overexpression of the majority of the cytokines and growth factors in the KO mice, especially for IL-6 (8.6-fold increase in the KO mice versus the control), IL-5 (5.8-fold increase), IL-12p70 (3.8-fold increase), CXCL13/BLC (2.7-fold increase), and G-CSF, CCL2/JE/MCP-1, TARC, and TIMP-1 (~1.7-fold increase for these 4). By comparing the KO and control groups for cytokine expression, the obtained dot blot values were similar to the outline obtained by the xMAP array analysis.

Proteome profiler analysis confirmed that a set of cytokines, chemokines, and growth factors was overexpressed in the plasma of the CAV1^−/−^ mice compared with that of the control mice, with significant differences for CXCL13/BLC, G-CSF, GM-CSF, CCL1/I-309, IL-3, and CXCL10/IP-10 (*p* < 0.05).

At this point in our study, we concluded that the CAV1^−/−^ mice are characterized by a low-grade systemic proinflammatory status.

### 3.3. Nonspecific Stimulation of the Lymphocytes of KO Mice with PHA and ConA

CAV1-KO mice were previously reported to show no changes in the percentages of lymphocyte subpopulations [27]; therefore, we used whole peripheral blood to initiate cell cultures and treated them with lymphocyte-targeting stimulants, that is, concanavalin A (ConA) and phytohemagglutinin (PHA). Using whole peripheral blood was reported as a valid method to assess cytokine production [25, 28]. We assessed whether the lymphocytes' response to stimuli is modified by the chronic inflammatory milieu. We found that upon stimulation with ConA or PHA, the production of cytokines/chemokines and growth factors increased, showing that even if these cells are derived from a medium abundant with proinflammatory cytokines, their response has not reached saturation.

**Figure 3:**
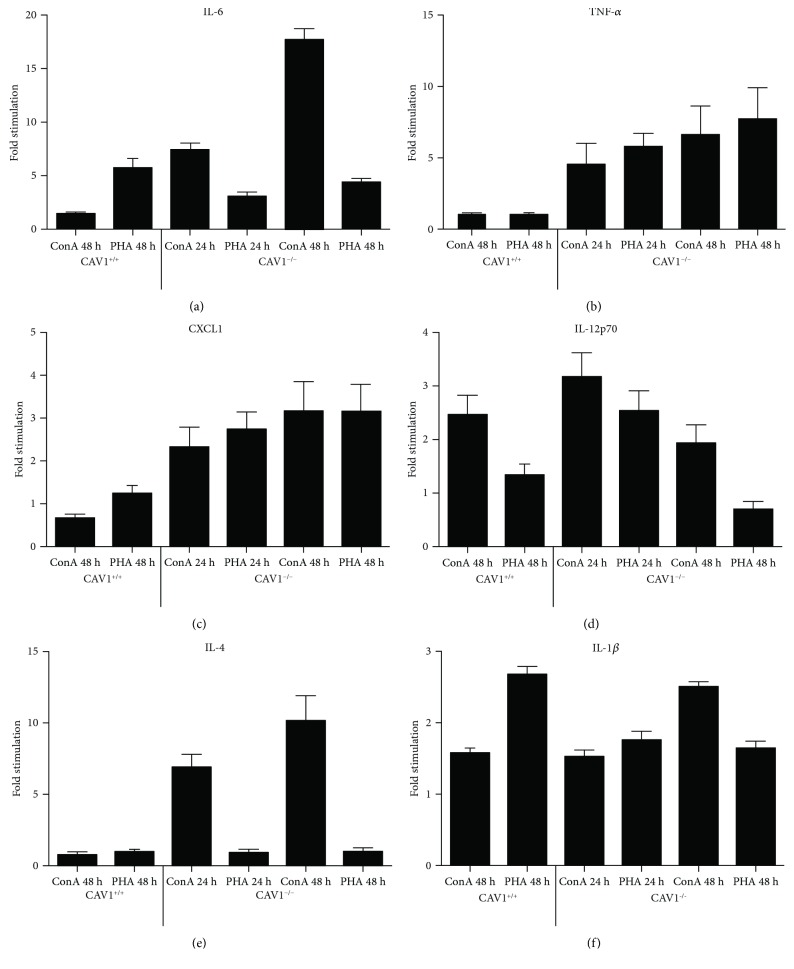
Cytokine production by lymphocytes upon nonspecific stimulation. Cytokine levels in the cell culture media of whole blood cells, treated with nonspecific lymphocyte stimuli at different time points after stimulation, as assessed by the xMAP multiplex assay: IL-6 (a), TNF-*α* (b), CXCL1 (c), IL-12p70 (d), IL-4 (e), and IL-1*β* (f). The data represent the fold modification of the cytokines from the CAV1^−/−^-derived cells versus the controls.

Overall, we noticed an activated status of the CAV1^−/−^ lymphocytes, characterized by an increased response to PHA and ConA stimulation by IL-6, TNF-*α*, CXCL1/KC, IL-4, and IL12p70, while IL-1*β* did not show the same trend (Figures 3(a) and 3(f)).

Expression of IL-6 in the CAV1^−/−^ lymphocytes increased at 24 h (7.4-fold compared to the control) and 48 h (17-fold versus the control), following ConA stimulation (Figure 3(a)).

Although TNF-*α* secretion was not inducible in the control lymphocytes, it increased in the CAV1^−/−^ lymphocytes upon stimulation. The relative increase was 2.8-fold and 7-fold, at 24 h and 48 h, respectively, for ConA stimulation and 5.8-fold and 14-fold, at 24 h and 48 h, respectively, for PHA stimulation (Figure 3(b)). CXCL1/KC was also overexpressed to 1.8-fold and 3.6-fold, at 24 h and 48 h, respectively, for ConA stimulation and to 2.7-fold and 5-fold, at 24 h and 48 h, respectively, for PHA stimulation (Figure 3(c)).

Expression of IL-12p70 in the CAV1^−/−^ lymphocytes increased, but only for the first 24 h after ConA stimulation (3-fold compared with the control), as well as after PHA stimulation (2.5-fold compared with the control) (Figure 3(d)).

IL-4 expression was also elevated in a time-dependent manner upon stimulation in the CAV1^−/−^ mice. Thus, 24 h after ConA stimulation, we observed an increase of almost 7-fold compared to the control, and at 48 h, the increase was 10-fold (Figure 3(e)). Similar to the control lymphocytes, the CAV1^−/−^ lymphocytes were nonresponsive to PHA stimulation.

For IL-1*β*, the response of the CAV1^−/−^ lymphocytes was time-dependent following ConA stimulation (1.5-fold and 2.5-fold compared to the control at 24 h and 48 h, resp.) (Figure 3(f)).

In conclusion, the circulating levels of the proinflammatory cytokines could generate a chronic inflammatory status; additionally, the lymphocytes of these mice are readily responsive to stimuli, further contributing to the inflammatory status, which can be a useful tool for future studies of the tumor environment or other low-grade chronic inflammatory diseases.

## 4. Discussion

CAV1 has been repeatedly linked to cancer progression, either as a tumor suppressor, as its absence is associated with a poor prognosis [29] (e.g., aggressive prostate cancer [30, 31], breast cancer [32], and gastric cancer [33]), or as a tumor and metastasis promoter [34–38]. CAV1-KO mice have been used extensively as a model to investigate tumor-related mechanisms, such as tumor growth, pathologic angiogenesis, and tumor invasion [26, 39]. In addition, CAV1-KO cells also activate inflammation-related signaling pathways (e.g., Akt signaling, TLR4 signaling, and ERK signaling), resulting in the production of proinflammatory cytokines, chemokines, and extracellular matrix remodeling enzymes [40]. Less is known about the contribution of CAV1 to the inflammatory milieu. Thus far, studies on CAV1 and inflammation have focused on organ-specific effects (lung [41, 42], colon [43, 44], and eye [45]). Additionally, different subsets of leukocytes derived from CAV1-null mice have been analyzed in terms of response to either parasitic [46] or bacterial infection [27]. Also, the ability of lymphocytes from CAV1-null mice to induce a humoral [47] or cytotoxic immune response [48] has been reported. However, studies have not focused on systemic inflammation in the absence of immune triggers.

CAV1 has also been linked with oxidative stress, in a dual manner. On the one hand, CAV1 was shown to be a “critical determinant” of oxidative stress balance. Using the same CAV1-null mouse model, Shiroto et al. found that the redox stress plasma biomarker 8-isoprostane was elevated in the blood of these mice and its involvement in oxidative stress was confirmed by knocking down CAV1 in endothelial cells in an in vitro model [49]. Furthermore, CAV1 was recently pinpointed as a target in cancer-related oxidative stress (reviewed in [50]). The relationship between CAV1, oxidative stress, and inflammation has been best studied in the tumor microenvironment, where loss of CAV1 was reported to lead to oxidative stress and to drive inflammation [51]. Moreover, loss of stromal CAV1 in a tumor model was proposed as a marker of inflammation and a predictor of poor outcome [52].

On the other hand, interesting data emerged about a direct interaction between CAV1 and nuclear factor erythroid 2-related factor 2 (NFE2L2/NRF2) [53], a transcription factor known as “master regulator of oxidative stress response” [54]. In this regard, inhibition of NRF2-mediated signaling by CAV1 activates the p53/senescence pathway [53] and inhibits antioxidant enzymes with antioxidant response element- (ARE-) dependent gene sequences [55].

In our study, we hypothesized that CAV1 has an effect on the systemic inflammation status. We addressed the involvement of CAV1 in enhancing the inflammatory response and creating a low-grade systemic inflammatory milieu. We verified the inflammation status by assessing the pro- and anti-inflammatory cytokine levels in the plasma of CAV1^−/−^ mice compared with that of control mice.

### 4.1. CAV1^−/−^ Mice Are Characterized by a Low-Grade Systemic Proinflammatory Status

To investigate whether the absence of CAV1 is related to a low-grade systemic inflammatory milieu, we performed a series of *in vitro* experiments examining the plasma protein levels of different cytokines in caveolin-1-deficient mice (CAV1^−/−^). To this end, we investigated the expression of circulating proinflammatory (IL-1*β*, IL-2, IL-6, IL-17, TNF-*α*, IL-8, IFN*γ*, CSF, and IL-12p70) and anti-inflammatory (IL-4, IL-10, and IL-13) members of the cytokine family in CAV1-KO mice, before the onset of any clinically overt tumors.

We showed that CAV1^−/−^ mice have enhanced plasma levels of a number of proinflammatory cytokines, including IL-1*β*, IL-2, IL-6, IL-12, and TNF-*α*, compared with those in the control mice (Figure 1). Inflammation is an important component of the tumor milieu and of the premetastatic niche, in which IL-6 is an important player [56, 57]. Our results are in agreement with recent findings that link CAV1 expression and IL-6 production. Lee et al. reported that the degradation of CAV1, via the ubiquitin/proteasome pathway, leads to TLR4 activation and the enhanced production of proinflammatory cytokines in bone marrow-derived macrophages [58]. *In vitro* silencing of CAV1 in mouse keratinocytes has been linked to STAT3 signaling activation, leading to increased expression of IL-6 [59]. Additionally, decreased expression of CAV1 in monocytes from diabetic peripheral neuropathy patients was negatively correlated with IL-6 and TNF-*α* plasma levels [60]. Taken together, these results support the fact that CAV1 expression is negatively correlated with IL-6 levels.

Weiss et al. also correlated the loss of CAV1 with increased TNF-*α* and other proinflammatory cytokines in a mouse colitis model [61].

Additionally, we found elevated levels of the anti-inflammatory cytokine IL-4 in the CAV1^−/−^ mice. However, these levels did not increase to the same extent as IL-6, as indicated by the IL-6 : IL-4 ratio of ~1 in the control group and 1.67 in the CAV1^−/−^ mice. One could speculate that IL-4 increases in the CAV1^−/−^ mice as a systemic reaction to compensate for the increase in the proinflammatory cytokines (IL-6 and TNF-*α*), but further investigation would be needed to validate such a mechanism.

The significant increase in IL-6 was confirmed by the Proteome Profiler, along with that in TNF-*α* and IL-12p70 (Figure 2). However, not all of the tested cytokines showed a modification of their circulation levels, compared with those in the control. As CAV1 has been previously described as an inhibitor of cell signaling, a loss of function can be correlated with the activation of different signaling pathways that result in subsequent cytokine production [51]. The significant increase in IL-6 reported in our study correlates with the activation of the JAK2/STAT3 signaling pathway reported by Yuan et al. in the lung endothelium of CAV1-KO mice [62], which was involved in *IL-6* gene transcription.

### 4.2. CAV1^−/−^ Lymphocytes Produce Enhanced Levels of Cytokines upon Stimulation

Endothelial cells and lymphocytes are common cellular sources of cytokines, and it has been demonstrated that endothelial inflammation is suppressed by CAV1 under physiological conditions [63]. Although uncontrolled inflammatory responses have been reported previously in relation to the loss of CAV1 [64], most of these studies have addressed the involvement of the lung endothelium [20, 21]. Owing to the abundance of caveolae in endothelial cells, these cells were the main target of CAV1 inflammation studies [10, 21, 65, 66]. To complement those studies, we addressed the contribution of other cells, namely, circulating lymphocytes, to cytokine production. Lymphocytes were initially considered negative controls for CAV1 expression, as they do not form caveolae, unless transfected with CAV1 [67]. Caveolin-1 has been detected and reported in leukemic cells [68, 69], possibly in conjunction with its involvement in cancer, and may reflect tumorigenic changes. We tested the response of CAV1-KO lymphocytes to nonspecific stimulation, which was quantified by the cytokine output in cell culture, as a possible tool to study low-grade inflammation. Our results showed that CAV1-KO lymphocytes are responsible for the production of various types of pro- and anti-inflammatory cytokines, depending on the type of stimulation and exposure time (Figures 3(a) and 3(f)). These results are supported by a number of studies reporting the role of CAV1 in primary T cells [48] and splenic B cells stimulated with LPS [27], as well as a recent report underlining the involvement of CAV1 in the regulation of B cell tolerance [47].

## 5. Conclusions

A CAV1-KO mouse model has been intensely used as a tool to study endothelial dysfunction, as well as tumor biology, owing to the increased susceptibility of these mice to cancer [70]. We hypothesized that CAV1 loss could also be involved in inflammation, which is a common feature of many pathologies, from cardiovascular diseases to tumor development. We demonstrated the existence of a low-grade systemic inflammatory milieu, characterized by moderately increased plasma levels of IL-6, TNF-*α*, and IL12-70p. Circulating lymphocytes of the CAV1^−/−^ mice were overresponsive to stimuli, indicating that these cells may contribute to the maintenance of this low-grade systemic inflammatory environment. Lymphocytes could also prove to be a useful tool to assess anticancer therapies that target inflammation. Our findings showed that CAV1-KO mice can also be used as an *in vivo* model for studying inflammation and could serve in the assessment of the anti-inflammatory effect of potential novel therapies. Given the strong association between inflammation and cancer [71–73], CAV1-KO mice may be useful for studies focusing on the intricate connections between inflammation and cancer. Finally, it could be added that CAV1, besides being a tumor suppressor, can also act as an inflammation suppressor that can be considered in the studies on CAV1-null tumors.

## Data Availability

The data used to support the findings of this study are available from the corresponding author upon request.
